# Factors affecting retention of veterinary practitioners in Ireland: a cross-sectional study with a focus on clinical practice

**DOI:** 10.1186/s13620-022-00222-9

**Published:** 2022-06-07

**Authors:** Eoin G. Ryan, Stephen H. Beatty, Elizabeth Gray, Niamh Field, Rory Liston, Victoria Rhodes, John Donlon

**Affiliations:** 1 Progressive Veterinary Network (PVN), Dublin, Ireland; 2 School of Veterinary Medicine, University College Dublin, Belfield, Dublin 4 Ireland; 3 Teagasc, Moorepark, Fermoy, Co. Cork Ireland

**Keywords:** Retention, Recruitment, Veterinary employees, Sustainability, Salary, Work-life balance, Gender equality

## Abstract

**Background:**

Retention of veterinary practitioners has arisen as a significant problem in recent years in Ireland. No prior Irish peer-reviewed publications have addressed this problem. An online questionnaire was available through social media and via email to Irish vets from January to November 2019. The aim of this survey was to ascertain the factors contributing to the problem of vet retention in Ireland.

**Results:**

A total of 370 eligible responses were received. The median age of respondents was 31 and the gender balance was 250 females (68%) to 118 males (32%). The majority of respondents worked in clinical practice 322 (89%), with 138 (42.8%) in mixed practice, 115 (35.7%) in small animal practice, 49 (15.2%) solely with farm animals and 20 (6.2%) in equine practice.

Fifty-four percent of respondents described themselves as likely to be leaving their current job within two years and 32.8% as being likely to leave the profession. In total, 44 variables were assessed by univariate analysis and 27 variables were significantly (*P* < 0.05) associated with the likelihood of a respondent leaving their current job within 2 years (LCJ2), as a proxy measure of the problem of retention. All variables significant on univariate analysis at *P* < 0.2 were included in a multivariable logistic regression model. Factors associated with LCJ2 included satisfaction with work-life balance (Odds Ratio (OR) 0.33); satisfaction with working hours (OR 0.2); number of years qualified (OR 0.91); position as a practice owner/partner/director (OR 0.15); and log_10_salary (OR 0.03).

Four variables were retained in a separate multivariable linear regression model as significant (*P* < 0.05) predictors of log_10_salary. Log_10_salary increased with years qualified. Males had an increased salary compared to females irrespective of years qualified. Part-time employees, vets on maternity leave or postgraduate vets had a lower log_10_salary. Compared to veterinary employees, self-employed or locum vets had a higher log_10_salary.

**Conclusions:**

Veterinary employers should consider salary, working hours and the facilitation of a good work-life balance in order to successfully retain veterinary employees. The significant difference in salaries currently offered to male and female vets, and the high percentage of respondents considering leaving the profession, are important findings and warrant further investigation.

**Supplementary Information:**

The online version contains supplementary material available at 10.1186/s13620-022-00222-9.

## Introduction

There have been concerns among veterinary practice owners in Ireland in recent years with respect to the difficulty they are experiencing in recruiting and retaining veterinary practitioners for clinical practice. In the Veterinary Practice Survey Report 2021–22, which is an annual report published by HLB Sheehan Quinn, 60% of respondents indicated that their practice was experiencing staff shortages [1]. The problems of recruitment and retention of veterinary practitioners is occurring even though the number of registered veterinary practitioners with the Veterinary Council of Ireland (VCI) has been steadily increasing and in 2021 reached 3222 individuals [2]. In the June 2021 issue of the Veterinary Ireland Journal, there were 51 available jobs for veterinary practitioners in Ireland [3]. This represents approximately a 40% increase compared to June 2016 when there were 37 available positions advertised [4]. In their recent 2019–2020 and 2020–2021 Veterinary Practice Survey Reports, HLB Sheehan Quinn found that recruitment was highlighted as being the biggest challenge facing veterinary practices today [5, 6]. Similar trends and difficulties in recruiting are seen in the UK [7–9] and in the United States [10].

Veterinary practitioners perform a crucial role in the fabric of society through their role as custodians of animal health and welfare, safeguarding the food chain and through their contributions to scientific research and academia. The emergence of the challenge of antimicrobial resistance has highlighted the importance of the veterinary practitioner in the whole area of One Health, One Welfare. Anecdotally, there has been an increase in the number of veterinary practitioners working in companion animal practice to meet the steady rise in companion animal numbers seen in this country. There remains a consistent demand for farm animal and equine practitioners. Despite the availability of work and jobs in Ireland, a serious recruitment and retention problem has arisen. This appears to be a more significant problem for farm animal practices, similar to the UK [8] and the US [10].

Recruitment difficulty could lead to financial losses for practice owners. In a publication on employee turnover in businesses, negative consequences associated with employee turnover included costs both tangible, like recruitment, selection, training and production loss, and intangible costs like moral impact, workload impact and team performance disruption [11]. The economic costs of turnover can be very significant for businesses [12]. For example, Merck and company, the pharmaceutical giant, has estimated that its turnover costs are between 150 and 250% of the employee’s annual salary [13]. High staff turnover can also result in a lack of continuity of care which can be a cause for clients to seek veterinary care elsewhere. Experienced veterinary practitioners are an essential part of the veterinary team as they can contribute greatly to a high standard of care on offer and can assist with training and mentorship of new and recent graduates. Therefore, the ability to retain a skilled and experienced veterinary workforce will have a direct impact on animal welfare and food production in Ireland.

In a recent UK publication, a number of factors were found to be affecting recruitment and retention of vets in the UK veterinary profession [7]. Vets who were recently qualified, on lower salaries and female were more likely to leave their current position. The most commonly cited reasons to leave a current employment position included work-life balance, management factors and salary. According to Hagen et al., respondents most disliked dealing with people, deficiencies in work-life balance and the physical/emotional impacts of the job. Respondents in that study indicated that they would most like to change the hours worked, team aspects and management. The authors concluded that the current retention crisis in the UK was due in part to the differing requirements between modern-day veterinary employees, their employers, the public and the profession [7].

Retention of vets in farm animal practice has been identified as a key issue in the sustainability of veterinary businesses and livestock health. In a UK cross-sectional study, Adam et al. identified factors influencing the retention of vets in farm animal practice [8]. Working in a practice where accommodation was provided and an increasing number of years since graduation were associated with significantly lower odds of remaining in farm animal practice, while working in a practice where staff appraisals were carried out; coming from a family with a commercial farm; spending more time on farm work and being on call with an experienced vet in the first job after graduation increased the odds of remaining in farm work [8].

It is likely that factors influencing the retention and recruitment of veterinary practitioners in farm animal practice may vary between different countries. For example, one of the largest barriers to retention and recruitment of veterinarians in the US is student debt [10]. In contrast, from an Irish perspective, while student debt is a big consideration for veterinarians that have completed a graduate entry degree course, or that have graduated from a fee-paying institution, student debt is usually less of a factor impacting on graduates of the 5-year veterinary medicine degree programme from University College Dublin (UCD). In a recent publication from the US Council for Agricultural Science and Technology (CAST), the challenges of rural life such as lack of social and cultural opportunities, lack of access to jobs for spouses, and childcare were of particular concern for recruitment and retention of farm animal veterinarians in US rural communities [10]. This was compounded by rural practices’ attributes such as long workdays and high on-call demands.

The demographic of the available veterinary workforce may also be evolving. According to the Federation of Veterinarians of Europe (FVE), the majority of European vets are now under 45 [14]. With a younger workforce comes generational differences in workplace values. More vets are now seeking less traditional veterinary roles e.g. management roles and work in the pharmaceutical industry [14]. Emigration is proving a popular option for veterinary practitioners who are moving to the UK, Canada and Australia to seek employment. As is seen in North America and across Europe [14], there is a feminisation of the veterinary profession occurring in Ireland. The impacts of feminisation of the profession must be considered as part of research into retention and recruitment issues, e.g. due to the increased likelihood of women looking to change jobs [7].

The objectives of this cross-sectional study were to ascertain the main factors contributing to the problem of veterinary retention, particularly in general practice, in Ireland, and to use this data to begin the process of addressing the important risk factors with a view to optimising the future sustainability of the Irish veterinary profession.

## Materials and methods

### Study design

A cross-sectional study design was used to identify the main factors associated with recruitment and retention of veterinary practitioners in Ireland. The research proposal was approved by the Human Research Ethics Committee – Sciences (HREC-LS) in UCD before data collection was commenced (LS-E-18-224-Ryan).

### Questionnaire

Data was collected via a web-based questionnaire using Qualtrics software version November 2019. Copyright© 2019, Qualtrics [15]. The survey questions asked are displayed in Additional File 1. An online questionnaire was shared with existing members (employees throughout the veterinary profession) of the Progressive Veterinary Network (PVN) using email, and on social media, through Facebook (Veterinary Voices Ireland Facebook page – used by a mix of veterinary employers and veterinary employees) [16]; PVN Facebook page [17] and via the PVN website [18]. While all VCI registrants were not exposed to the questionnaire, the online approach taken allowed us to survey a wide population of the veterinary profession registered as practicing or working in Ireland. All respondents were asked to give their approval for their response data to be used for the purposes of publications and to inform the veterinary profession in relation to the factors influencing the problems of retention and recruitment of veterinary practitioners in Ireland. The online survey was open for responses from 09/01/2019 to 11/11/2019.

### Participants

The target population were all veterinary practitioners registered with the Veterinary Council of Ireland (VCI) working in Ireland. To be eligible, the target population had to give prior consent for publication. Method of recruitment was via self-selection following online and email advertisements. As of January 2019, there were 2693 veterinary practitioners registered with the VCI and, in total, there were 2956 veterinary practitioners, 57% male and 43% female, on the register at the end of 2019 [19]. More detail on the age demographics of veterinary practitioners registered with the VCI in 2019 is displayed in Table 1. Using a confidence level of 95% and population proportion of 0.5, a desired sample size of 336 was calculated as sufficient to yield results representative of the target population [20]. Due to the anonymity of the survey, it was possible that a respondent could answer more than once. To prevent this, restrictions were placed on use of the same IP address and IP addresses were checked to prevent doubling of answers.

**Table 1:** Age-related demographic data of veterinary practitioners registered with the Veterinary Council of Ireland in 2019 (VCI Personal Communication, 2022)

Age Range by Decade of Birth	% of Veterinary Practitioners
1920s	0.1%
1930s	2%
1940s	7%
1950s	12.4%
1960s	15.5%
1970s	22%
1980s	26%
1990s	15%

### Variables

Quantitative variables included age, years graduated, salary, days per week worked, hours per week worked, annual leave, number of jobs worked, longest duration worked and length of stay in the first job (Table 2). No quantitative variables were grouped.

**Table 2:** Descriptive results of study population variables (*n* = 370)

Category (***n*** = respondents answered)	Answer	%	n
Employment Status	Full time	90.8	327
	Part time	7.2	26
	Unemployed	0.3	1
	Locum	1.1	4
	Postgraduate	0.6	2
	Answered	100	360
	Missing data		10
Length in last job	< 1 year	15.5	52
	1–2 years	25.4	85
	2–3 years	14.6	49
	3–4 years	10.4	35
	> 5 years	14.9	50
	Still in first job	19.1	64
	Answered	100	335
	Missing data		35
Longest duration in one job	Mean (Years) (SD)	5.2	320
	Missing data		50
Satisfaction in current job	Extremely satisfied	16.6	55
	Somewhat satisfied	47.9	159
	Neither satisfied nor dissatisfied	8.7	29
	Somewhat dissatisfied	20.8	69
	Extremely dissatisfied	6.0	20
	Answered	100	332
	Missing data		38
Satisfaction with work-life balance	Satisfied	37.5	124
	Neither satisfied nor dissatisfied	9.7	32
	Dissatisfied	52.9	175
	Answered	100	331
	Missing data		39
Likelihood of staying in Ireland	Extremely likely	70.2	233
	Somewhat likely	17.5	58
	Neither likely nor unlikely	3.3	11
	Somewhat unlikely	6.9	23
	Extremely unlikely	2.1	7
	Answered	100	332
	Missing data		38
Likelihood of leaving vet profession in next 5–10 years	Extremely likely	10.3	34
	Somewhat likely	22.5	74
	Neither likely nor unlikely	11.9	39
	Somewhat unlikely	16.4	54
	Extremely unlikely	38.9	128
	Answered	100	329
	Missing data		41
Likelihood of looking for a new job in the next 2 years	Extremely likely	32.4	107
	Somewhat likely	21.2	70
	Neither likely nor unlikely	8.5	28
	Somewhat unlikely	16.4	54
	Extremely unlikely	21.5	71
	Answered	100	330
	Missing data		40
Reason for leaving current position (Pick 3 reasons) Top 5 displayed.	Work-life balance	51.4	127
	Salary	36.8	91
	Family	26.7	66
	Working hours	26.3	65
	OOH Rota	25.5	63
	Answers	100	247
	Missing data		-
Future aspirations if leaving current position (Multiple answers permitted)	Same type of work, but different employer	47.6	121
	Different work, but still in veterinary field	27.2	69
	Take a break for family or travel	14.2	36
	Leave the veterinary field	10.2	26
	Partnership	0.4	1
	Retirement	0.4	1
	Answers	100	254

### Statistical analysis

Questionnaire data collated in Qualtrics software (Qualtrics, Provo, UT). Copyright© 2019, Qualtrics [15] was downloaded to Microsoft Excel [21]. The data were then imported into R version 3.6.2 [22] for further data processing and statistical analysis. All data manipulation was conducted using the “dplyr” package in R [23]. Missing data was not imputed and was treated as an N/A.

Preliminary descriptive statistics were performed on all of the variables to gain a basic understanding of the study population. For continuous variables, mean, median and interquartile range were calculated. For categorical variables, counts and percentages were calculated. Two separate models were created, the first to determine the factors associated with the likelihood of leaving a current job within 2 years (LCJ2 model), and the second model examining the predictors of salary within the survey population (Salary model). Continuous data were first visualized using histograms to ensure they were normally distributed. The salary data set was not normally distributed and was transformed by log_10_. Ordinal data were treated as categorical data.

### LCJ2 model

All variables were first screened in a univariate analysis. Variables with a *P*-value < 0.2 were offered to a multivariable logistic regression model. Variables were added to the model in a forward stepwise manner in order of their univariate P-value with variables with the lowest P-value added first. After the addition of each variable, the *P*-values for the remaining variables were recalculated, and variables with *P* > 0.05 were excluded from the model.

### Salary model

All variables were first screened in a univariate analysis. Variables with a *P*-value < 0.2 were offered to a multivariable linear regression model. Variables were added to the model in a forward stepwise manner in order of their univariate P-value with variables with the lowest P-value added first. After the addition of each variable, the *P*-values for the remaining variables were recalculated, and variables with *P* > 0.05 were excluded from the model.

## Results

**Fig. 1:**
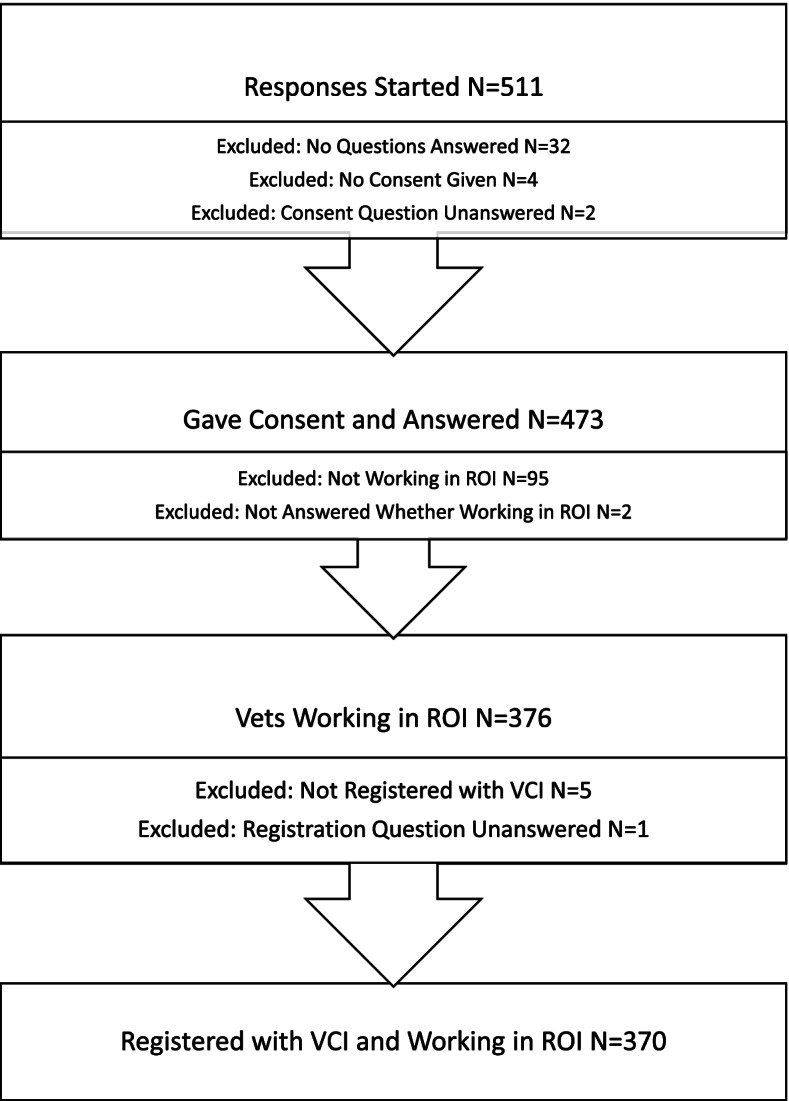
Numbers of participants at each stage of recruitment for survey analysis

The numbers of survey responses received and included in the analysis are shown in Fig. 1. In total, there were 370 respondents who either fully or partially completed the questionnaire and upon whose responses descriptive and statistical analyses were carried out.

### Descriptive data

The median age of the survey respondents was 31 (range 22–72 years) and the gender balance was 248 females (67.4%) to 118 males (32.1%). The majority of respondents (298) received their veterinary education in University College Dublin (81.4%), with 38 (10.4%) respondents having attended Szent Istvan University, Budapest and the remainder attending UK and other European universities. Of 321 respondents, 249 (77.6%) described themselves as associate veterinarians or employees of a practice; 40 (12.5%) described themselves as practice owners/partners and 8 respondents described themselves as Manager or Clinical Director. Of 358 respondents, the average length of time since qualification as a veterinarian was 8.7 years (7.8 years for female vets and 10.6 years for male vets) and the median length of time was 7 years. While all 26 counties of the Republic of Ireland were represented in the questionnaire results, of 355 survey respondents, 58 worked in Dublin (15.8%), 51 worked in Cork (13.9%), with Tipperary (7.4%) and Galway (7.1%) the next highest. Of 365 respondents, 325 indicated that they were working in clinical practice (89%), 28 respondents were providing government veterinary services (7.4%) and the remaining 15 respondents worked between academia, the pharmaceutical industry and as temporary veterinary inspectors (TVI’s, i.e. part-time government contracted meat inspectors). With respect to those respondents working as full-time vets in clinical practice, 138 were working in mixed practice (42.7%), 115 were working in small animal practice (35.6%), 49 were working solely with farm animals (15.2%), 20 respondents were working solely in equine practice (6.2%) (Additional File 2).

In terms of employment status, 90.8% of respondents were in full-time employment, 7.2% in part-time employment and 2.0% with another status of employment such as locum or maternity leave (Table 2). Of those in clinical practice, 78.3% were employees, 14.6% were practice owners/partners/clinical directors and 7.1% were self-employed. 19.1% of respondents were still in their first job, and the longest duration that respondents were in one job was on average 5.0 years. 64.5% of respondents were either somewhat satisfied or extremely satisfied with their current job, while 26.8% were either somewhat dissatisfied or extremely dissatisfied (Table 2). The majority of respondents (53.6%) described themselves as likely to be looking for a new job within two years, while 37.9% of respondents were likely to remain in their current job. The likelihood of respondents leaving the profession within the next 5 to 10 years is also illustrated in Table 2. It can be seen that 32.8% of respondents described themselves as likely to leave the profession, while 55.3% of respondents were unlikely to leave the profession. Only 37.5% of respondents described themselves as satisfied with work-life balance. In contrast, 52.9% of respondents were dissatisfied with work-life balance (Table 2).

### Risk factors associated with the likelihood of leaving current job within two years (LCJ2 model)

In total, 44 variables were assessed by univariate analysis (Additional File 3) and 27 variables were found to be significantly associated with the likelihood of a respondent leaving their current job within 2 years (*P* < 0.05).

Factors that contributed to current job satisfaction, and less likelihood of leaving a current employment, included satisfaction with the number of hours respondents were required to work (*p* < 0.001), the amount of out of hours worked (*p* = 0.005), satisfaction with benefits coming with the job (*p* < 0.001), including a contribution to continuing professional development (CPD) (*p* < 0.001), the provision of maternity/paternity leave (*p* = 0.003) and a provision for sick leave as needed (*p* < 0.001). An extended 1 in 5 rota for on-call (*p* = 0.007) and out of hours (OOH) work (*p* = 0.005) was associated with enhanced job satisfaction and a reduced likelihood of leaving the current job. Similarly, if respondents were satisfied with their current salary, they were less likely to consider leaving their current job within 2 years (*p* = 0.002). Respondents that described themselves as practice owners/partners or directors were less likely to leave their current job (*p* < 0.001). Similarly, if a respondent had spent 5 years or more in their previous job, they were more likely to remain longer in this current position (*p* < 0.001). Respondents that were satisfied with their work-life balance were much less likely to move on to another job within 2 years (*p* < 0.001). Respondents that were less likely to leave the veterinary profession within 5–10 years were also less likely to leave their current position (*p* < 0.001). Male veterinary practitioners were less likely to move on from their current job within two years than female veterinary practitioners (*p* < 0.001). Veterinarians that were already working in state jobs, e.g. for the Department of Agriculture, Food and the Marine (DAFM), were found to be less likely to move on from their current job (*p* < 0.001). On the other hand, veterinary practitioners that did not have an interest in leaving practice for a position with DAFM were likely to stay in their current position (*p* = 0.003), as were veterinary practitioners working in the Kildare and Meath region (*p* = 0.002).

Some factors were found to significantly increase the likelihood of a respondent moving on to another job within two years. These factors included family reasons and reasons of poor work-life balance (*p* = 0.04); a lack of annual leave provision and CPD allowance (*p* = 0.02); a desire to travel (*p* = 0.01) and having no strong feelings about wanting to preferentially stay and work in Ireland (*p* = 0.04).

Other factors were found to have both positive and negative associations on a respondent’s decision making on whether to leave their current job within two years. These factors included years qualified (*p* < 0.001) and age (*p* < 0.001), with younger, recently qualified veterinary practitioners more likely to move on to a new position, while older veterinary practitioners graduated for a longer period were more likely to remain in their current job. Log_10_salary was found to significantly influence respondent decision making (*p* < 0.001), with veterinary practitioners on lower salaries more likely to leave their current employment within two years, while veterinary practitioners on higher salaries were less likely to move on. Recently qualified veterinary practitioners, that had only worked in a small number of jobs (*p* < 0.001), especially jobs of shorter duration, were more likely to move within 2 years to another job. All variables significant on univariate analysis at *P* < 0.2 were then included in a multivariable logistic regression model to look for variables significantly associated with the likelihood of leaving their current job within 2 years. In the end, 5 variables were retained in the logistic regression model as significant predictors of likelihood of leaving their current job within 2 years (Table 3).

**Table 3:** Results of the LCJ2 multivariable logistic regression model showing variables predictive of the likelihood of respondents leaving their current job within 2 years

LCJ2 Model Results	Estimate	Std. Error	z value	***P*** value	Odds Ratio	95% Confidence Intervals
**(Intercept)**	18.6211	6.1930	3.007	0.0026 **		
**Satisfied with work-life balance (Referent = Dissatisfied with work-life balance)**	−1.1067	0.4350	−2.544	0.0110 *	0.3307	(0.1410, 0.7756)
**Satisfaction with hours worked - Neither satisfied nor dissatisfied (Referent = Dissatisfied with hours worked)**	−1.6114	0.4509	−3.573	0.0004 ***	0.1996	(0.0825, 0.4831)
**Years qualified**	−0.0918	0.0294	−3.125	0.0018 **	0.9123	(0.8613, 0.9664)
**Position as practice owner/partner/director (Referent = Associate veterinarian/employee)**	−1.8829	0.5694	−3.307	0.0009 ***	0.1521	(0.0498, 0.4645)
**Log**_**10**_**salary**	−3.5344	1.3197	−2.678	0.0074 **	0.0292	(0.0022, 0.3876)

The multivariable logistic regression model showed that as satisfaction with work-life balance increased, the odds of respondents leaving their current job within two years was significantly reduced (*p* = 0.01). Similarly, compared to a veterinary practitioner that was dissatisfied with their working hours, there was reduced odds of a veterinary practitioner leaving their current job within 2 years if they were neither satisfied nor dissatisfied with their working hours (*p* < 0.001). For every one-year increase in years qualified, the odds of a respondent leaving their current job within 2 years was significantly reduced (*p* = 0.002). Also, if a veterinary practitioner was already a practice owner/partner/director, there was a significantly reduced odds of them leaving their current job within 2 years (*p* < 0.001). Lastly, for every one-unit increase in log_10_salary, there was a significantly reduced odds of a veterinary practitioner leaving their current job within 2 years (*p* = 0.007).

### Factors associated with salary: descriptive statistics and salary model

Of 343 respondents, the median gross salary was €50,000, with median female gross salary of €45,750 (*n* = 232) and median male gross salary of €60,000 (*n* = 110). The salary breakdown across different areas of the profession is displayed in Table 4. Vets working in clinical practice had a markedly lower median gross salary in comparison to veterinary practitioners employed by the Government or other areas of the profession.

**Table 4:** Median gross salary by area of the profession

Area of the Profession	No. Respondents	Median Gross Salary (€)	Inter-quartile range (€)
**Clinical practice (Overall)**	304	48,000	20,000
**Clinical practice (Male)**	93	54,000	21,300
**Clinical practice (Female)**	210	45,000	15,000
**Government veterinary services (Overall)**	26	62,000	40,375
**Government veterinary services (Male)**	15	68,000	40,500
**Government veterinary services (Female)**	11	56,000	8,528
**Other (academia, pharmaceutical, TVI, laboratory) (Overall)**	13	62,000	38,000
**Other (academia, pharmaceutical, TVI, laboratory) (Male)**	2	85,500	40,500
**Other (academia, pharmaceutical, TVI, laboratory) (Female)**	11	56,000	37,250
**Overall Median Salary**	343*	50,000	20,000
**Overall Median Salary (Male)**	110	60,000	29,500
**Overall Median Salary (Female)**	232	45,750	15,000

*One respondent in clinical practice did not declare their gender

Of those respondents working solely in clinical practice, the variation in median gross salary is displayed in Table 5. The median gross salary of an associate/assistant veterinarian or veterinary employee was €47,000.

**Table 5:** Median gross salary by type of employment amongst respondents working in clinical practice

Employment Position	No. Respondents	Male Gross Salary (€)	Female Gross Salary (€)	Median Gross Salary (€)	Interquartile range (IQR) (€)
**Associate Veterinarian/employee**	240	50,000 (*N* = 65) (IQR 18000)	45,500 (*N* = 175) (IQR 14500)	47,000	15,000
**Practice owner/partner/director**	44	75,500 (*N* = 20) (IQR 33750)	42,500 (*N* = 24) (IQR 24450)	55,000*	35,000
**Self Employed/Locum**	19	54,500 (*N* = 8) (IQR 35,000)	50,000 (*N* = 11) (IQR 38,750)	50,000	37,500
**Overall Median Salary**	303	54,000 (*N* = 93) (IQR 21,300)	45,000 (*N* = 210) (IQR 15,000)	48,000	

*One respondent did not reveal gender status

It can be seen in Table 6 that there was a variation in median gross salary between associate veterinarians/employees in different areas of clinical practice, as well as a difference in median gross salary by gender with respect to clinical practice area. There was a gender pay gap (GPG) of 10% between male and female associate veterinarians/employees working in clinical practice. For the purposes of this study, GPG was calculated by expressing the difference in median gross salary between genders as a percentage of median male gross salary. For all respondents (Table 4), clinical and non-clinical, with salary and gender data declared (*n* = 343), there was a gender pay gap with median male gross salary of €60,000 (*n* = 116) and median female gross salary of €45,750 (*n* = 242). This resulted in a difference in salary by gender of €14,250 (Table 4) which was equivalent to a gender pay gap of 23.8%.

**Table 6:** Median gross salary of associate veterinarians/employees by type of clinical practice

Clinical Practice	Female Gross Salary (€)	Male Gross Salary (€)	Median Gross Salary (€)
**Equine**	52,000 (*N* = 11) (IQR 19,000)	55,000 (*N* = 2) (IQR 5000)	52,000 (IQR 18,000)
**Farm Animal**	46,250 (*N* = 14) (IQR 17,250)	50,000 (*N* = 20) (IQR 15,000)	49,000 (IQR 15,750)
**Mixed**	45,000 (*N* = 76) (IQR 12,375)	46,000 (*N* = 35) (IQR 19,000)	45,000 (IQR 14,500)
**Small Animal**	46,500 (*N* = 74) (IQR 14,250)	54,000 (*N* = 8) (IQR 22,750)	47,500 (IQR 14,000)
**Overall**	45,000 (*N* = 175)	50,000 (*N* = 65)	47,000 (*N* = 240)

**Fig. 2:**
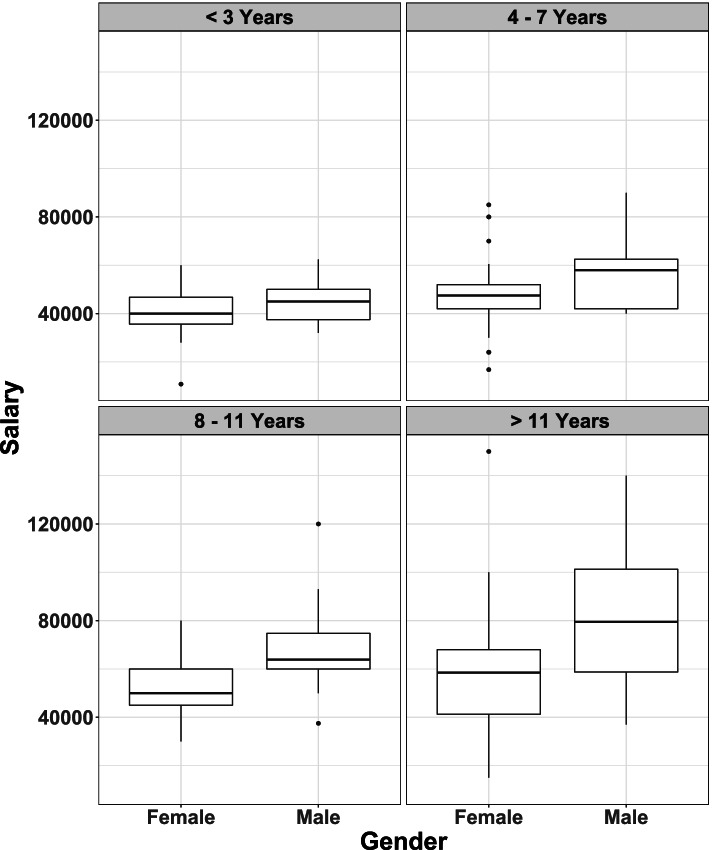
Comparison of salary of male (*n* = 110) and female (*n* = 230) respondents by years qualified

It can be seen from Fig. 2, that there was a difference in salary by gender with respect to years qualified, with male veterinary practitioners receiving a higher salary. This difference in salary between the genders appeared to become greater with increased years qualified.

**Fig. 3:**
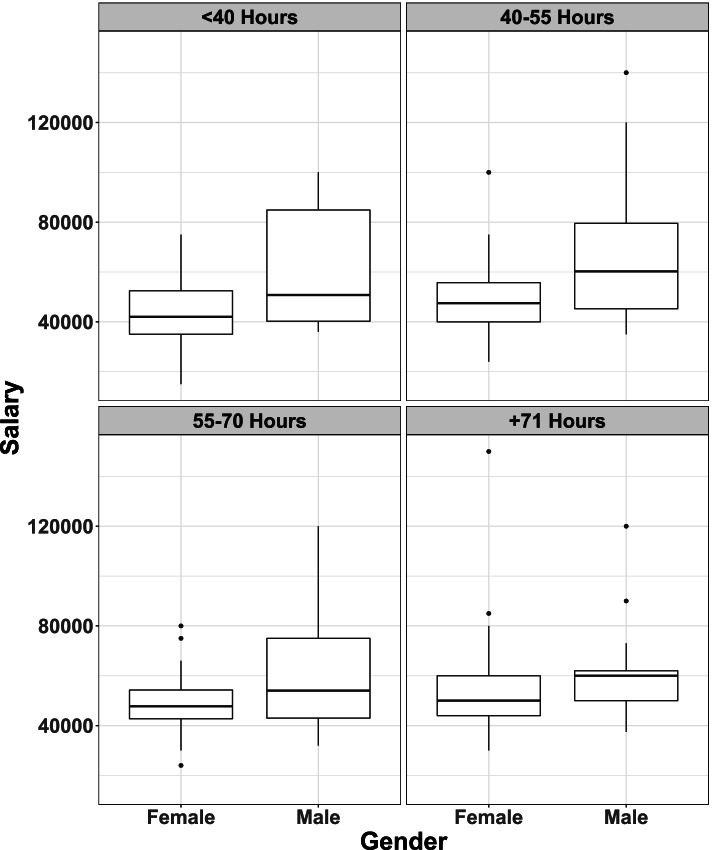
Comparison of salary of male (*n* = 109) and female (*n* = 225) respondents by hours worked per week

It can be seen from Fig. 3, that male veterinary practitioners received a higher salary irrespective of hours worked.

### Quantitative statistics

Univariate statistical analysis was carried out on 44 distinct variables and 26 variables were found to be significantly associated with log_10_salary at a *P* < 0.05 (Additional File 4).

On univariate analysis, being employed part-time, being unlikely to stay in Ireland, and having an opinion that income suffered from not having the possibility of temporary veterinary inspector (TVI) work at that time, were negatively associated with log_10_salary. All other variables were associated with a higher log_10_salary.

All variables significant to a confidence level of *P* < 0.2 on univariate analysis were then included in a multivariable linear regression model to identify those variables which significantly predicted log_10_salary. Five variables were retained in the Salary Model (Table 7).

**Table 7:** Multivariable linear regression model outcome showing predictors of Log_10_salary

Log_**10**_salary	Estimate	Std. Error	t value	***P*** value
**(Intercept)**	4.5573	0.0482	94.582	<2e-16***
**Years Qualified**	0.0074	0.0012	5.985	7.39e-09***
**Gender Male (Referent = Female)**	0.0536	0.0151	3.540	0.0005***
**Employment: Other**^**+**^ **(Referent = Employment Full-time)**	−0.2640	0.0621	−4.253	2.97e-05***
**Employment: Part-time employment (Referent = Employment Full-time)**	−0.2042	0.0312	−6.548	3.25e-10***
**Position: Self-employed/locum (Referent = Associate Vet/Employee)**	0.1191	0.0346	3.443	0.0007***

^+^ Other = Locum and postgraduates; *** variables with a P-value< 0.001

The salary model showed that there was a significant increase in log_10_salary with each year qualified (*p* < 0.001). Males had a significantly higher log_10_salary compared to females, irrespective of years qualified (*p* < 0.001). Compared to being a full-time employee, part-time employees had a significantly reduced log_10_salary (*p* < 0.001). Compared to being an associate veterinarian or employee, veterinary practitioners that were self-employed or doing locum work had a significantly higher log_10_salary (*p* < 0.001).

## Discussion

There has been a steadily worsening problem with recruitment and retention of veterinary practitioners in Ireland, particularly within clinical practice, over the last number of years. Similar trends and difficulties in recruiting and retaining vets have been described in the UK [7, 8] and in the United States [10]. Other health professions in Ireland, e.g. doctors, are also facing retention challenges [24]. The main aim of this study, which was the first of its type in Ireland, was to determine the factors influencing retention of veterinary practitioners in Ireland. This survey was open to all veterinarians registered with the Veterinary Council of Ireland in 2019. However, the majority of participants in this study were working in clinical practice (88.5%), which was similar to the demographics of the recent UK recruitment and retention paper (90.3%) by Hagen et al. 2020. New graduates, classified as veterinary practitioners qualified less than or equal to five years [25], accounted for 36% of survey respondents. In comparison to Hagen et al. [7], 68% of our respondents were female compared to 77% in the aforementioned UK study.

This study has highlighted a number of extremely important findings which shed light on the current problems of retention of veterinary practitioners within the Irish veterinary profession. The LCJ2 Model identified that the main considerations influencing the likelihood of veterinary practitioners leaving their current job within two years were work-life balance, number of hours worked and salary. Studies in Australia, Germany and the United Kingdom also found that work-life balance is a problem in their respective veterinary industries [7, 26, 27]. Work-life balance was one of the main reasons for veterinary practitioners planning to leave their employment position in these studies and was correlated with increased stress [7, 26]. Twenty-two percent of employers in one study believed that work-life balance contributed to employees leaving their position [7]. In our study, only 37.5% of respondents described themselves as satisfied with work-life balance. In the authors’ experience, many Irish veterinary practitioners, especially during their first five years, emigrate and work in numerous countries around the world. This trend among veterinary practitioners has also been mirrored in the Irish medical profession [28]. In a contemporary study, the retention of Irish trained hospital doctors was as much associated with the quality of the work experience afforded Irish trained doctors, as it was the quantity and composition of the workforce in which they participated [28]. The conclusions from a recent Icelandic study [29], found that a shorter working week was associated with workers experiencing significant increases in wellbeing and work-life balance, all while existing levels of service provision and productivity were at the very least maintained, and in some instances improved.

The LCJ2 model also showed that veterinary practitioners qualified longer, and particularly veterinary practitioners already established as practice owners/partners, were less likely to leave their current position of employment. A similar finding was highlighted in the study of Hagen et al. [7], where recently qualified vets were more likely to leave their current employment position. This UK study found family ties to be a strong determinant of whether a vet was likely to remain working in a particular job or area. Family ties and business commitments, as practice partners/owners, could also be reasonably assumed to explain this finding in our study. Indeed, family reasons and work-life balance were significant findings associated with the likelihood of leaving their current employment within two years on univariate analysis in our study.

A worrying finding from our study was the fact that 32.8% of respondents described themselves as likely to leave the profession. This is approximately double the percentage of veterinary surgeons (17.2%) described as planning to leave the profession in the future in the UK study of Hagen et al. [7]. The Irish veterinary profession should take cognisance of this apparent lack of job satisfaction among recent graduates and take action to minimise the haemorrhage of talent. While the majority of participants expected to apply for new positions within two years (53.6%), most of these respondents wanted to remain in the veterinary profession (53%) and live in Ireland (78%). This suggests that where improvements can be made in either work-life balance, financial gain or both, progress in retaining veterinary practitioners in their current positions could be achieved.

The LCJ2 model highlighted salary as a significant factor associated with the likelihood of veterinary practitioners leaving their current employment. In previous similar studies, salary was also cited as a common factor for leaving current employment in veterinary practice. A recent survey of vets in the UK found that vets who were female, on lower salaries, and those recently qualified were more likely to plan to leave their current position [7]. In that study the most common reasons given by respondents for leaving their current employment were, in order of frequency, work-life balance, management and salary. A survey of veterinary practitioners in the United States found that salary ranked third behind emergency work and time off as a reason for leaving rural practice specifically [30].

The Salary model in our study identified three significant factors which influenced salary levels among survey respondents. These included years qualified, gender and employment status (self-employed and locum work). We have already discussed how years qualified could be associated with the likelihood of a veterinary practitioner already having reached the status of partner/owner, which would likely also account for an increased salary amongst this cohort of respondents.

While not the aim of this study, a significant gender pay gap (GPG) of 10% was found between male and female veterinary associates /employees working in clinical practice only, accounting for working hours per week (see Table 6). This compared similarly to the documented GPG in Ireland across all professions in 2018 of 11.3% [31]. Our findings are similar to other countries such as the UK where the Society of Practising Veterinary Surgeons (SPVS) Salary survey 2020 [32], found a 15% GPG among veterinary surgeons. In contrast, the SPVS survey found that the GPG was much smaller in the group qualified < 15 years (3–6%). In the Federation of Veterinarians of Europe 2018 Vet Survey [14], female vets were paid on average 12% less than their male colleagues which shows that this issue is reflected at a European level. Even though the GPG between male and female veterinary associates/employees working in clinical practice in our study appears to be consistent with other professions, this should not disguise the fact that a gender pay gap undoubtedly exists within the Irish veterinary profession. This is a very disappointing finding and merits future attention by all stakeholders, including practice owners/partners, Veterinary Council of Ireland, Department of Agriculture, Food and the Marine (DAFM), other relevant government departments, and veterinary representative bodies. Interestingly, we saw an overall GPG of 23.8% when accounting for the total population of study respondents, and an even larger GPG between male and female practice owners/partners of 43.7%. Although there were only 46 practice partners/owners in our study population, this GPG is alarming and would certainly warrant further investigation to determine the significance of our finding. If this is a consistent finding in a larger study of practice partners/owners, this could potentially represent a barrier to women within the veterinary industry progressing professionally from a financial point of view.

Many factors are thought to contribute to a GPG such as education, hours worked and lack of affordable childcare, some of which may or may not apply to the veterinary industry in Ireland. In Ireland women account for 70% of the part-time workforce [33] and this is often cited as one of the main factors which could explain the GPG. In our survey, there was no evidence of either working hours or part time work contributing to the lowering of female salaries as the majority of participants were involved in full time employment. Currently, there are no legal obligations for veterinary employers to share information on the GPG of employees. The Gender Pay Gap Information Act 2021 [34] requires certain employers to share the differences in pay between male and female staff. However, this legislation does not apply to employers with less than fifty employees. Veterinary employers should take note of the greater attention on the issue of GPG at a national level.

There were some limitations with regard to our study. As with all study types, cross-sectional studies have their own underlying biases. These biases include selection bias, information bias and confounding. Selection bias occurs when the participants in the study differ systematically from those not selected. With respect to our study, there was a bias towards veterinary associates/employees and veterinary practitioners with internet access and more familiarity with social media (PVN website [18]; Facebook; Instagram). The median age of participants in our study was 31 years of age. This is slightly younger than the age distribution of the registered veterinary practitioner population in 2019 (Table 1). There was also a gender bias, with 67.4% of respondents in this study female compared to a gender distribution of 43% female in the registrant population in 2019. Information bias can include recall and detection biases. There can be a tendency for responses to be socially acceptable, rather than the truth when self-reporting [35]. However, this survey was anonymous in order to maximise factual answers. Confounding was dealt with during the statistical analysis of our findings.

The results of the statistical analysis from the survey support the findings that salary and work-life balance have a direct effect on retention of veterinary practitioners. These results are consistent with other studies published in similar areas. In order to maximize retention of veterinary employees, practice owners should focus on ways to optimise employee salary and work life balance.

With a large proportion of survey participants showing a desire for increased salary, veterinary employers should ensure that their veterinary employees are paid to industry standards and commensurate with their work, skills and expertise. At the current time of writing there is no salary guide for veterinary employers or employees in Ireland. This can create discrepancies amongst what veterinary practitioners earn for similar work performed. The authors believe that a salary guide would help standardize salaries thus allowing employees to judge their salary against the industry standard. SPVS in the United Kingdom provides members with results of an annual salary survey that details average salaries seen throughout the profession [32]. In North America, the American Veterinary Medical Association provides an online salary calculator for veterinary professionals [36]. This provides veterinary professionals with a benchmark from which they can negotiate salaries during the interview process. It may also help reduce the stigma associated with discussions around salary in veterinary medicine and provide students with a realistic salary expectation. Salary was found to be highly statistically significant in relation to the likelihood of veterinary practitioners seeking employment elsewhere. Therefore, this suggests that an increase in salary is inherently associated with the likelihood of registrants remaining in their current jobs. This is an important point for employers to consider, as it is likely that an increase in salary will lead to a reduction in turnover of veterinarians. An interesting finding in our study was the difference in salaries between veterinarians in clinical practice and those working in government positions, with government veterinarian salaries being 40% higher than those in clinical practice. This large gap may increase the desirability of government roles over clinical practice, and this may have contributed to the transfer of a considerable number of veterinary practitioners from clinical practice to roles with DAFM in recent years.

The majority of veterinary employees working in clinical practice worked between 50 and 70 hours per week, highlighting a work life balance issue in veterinary practice in Ireland. This was also echoed in a recent Irish survey, in which 43% of respondents worked greater than 50 hours per week [5]. A recent UK study [7] reported that the majority of participants worked 41–60 hours per week, which was lower than the 50–70 hours worked on average by respondents in our study. While the working hours referenced in our study included out of hours/on call hours, this was still considerably more than the average working week in Ireland of 39 hours per week and would call into question the likelihood of adequate break periods being made available to veterinary employees if they work a 50–70 hour week. Efforts should be made to ensure that veterinary employees are given the legal minimum rest period of 11 hours rest for every 24-hour working period, with defined break intervals during the working day, and the maximum legal average working hours of 48 hours per week as defined in section 15 [1] of the Organisation of Working Time Act, 1997 [37]. A work-life imbalance has been directly linked to burnout in human healthcare scenarios [38] and long working hours have been associated with poor mental health [39]. The UK Vet Futures 2015 publication entitled ‘Taking charge of our future: A vision for the veterinary profession for 2030’, identified health and wellbeing and achieving a good work life balance among the top goals for the veterinary profession [9]. The UK ‘Vet Futures’ initiative provides a template which the Irish Veterinary Profession could benefit from exploring [9].

Within our survey population, 53.6% of veterinary practitioners were considering leaving their current role within the next two years. Given that finding, it would be prudent to make suggestions of how we, as a profession, could practically and positively influence those factors identified as impacting the retention of veterinary practitioners in Ireland. The introduction of novel Out of Hours (OOH) rotas may help provide veterinary employees with more free time between their on-call duties. Likewise, the availability of dedicated OOH veterinary service providers would alleviate the need for working veterinary practitioners to be included in an OOH rota altogether. The authors are aware of the practicalities of providing such a service and realise that it may be limited to practice groups in an urban setting. Improvements to workplace culture such as making sure staff take their lunch breaks, finishing clinics on time and respecting employees’ free time may seem like small steps but can make a difference in enhancing work-life balance. In relation to salary within the veterinary profession there are many complexities and potential factors to consider. A review of current veterinary practice structures, business models and remuneration packages are necessary to ensure sustainability of the veterinary profession in Ireland. It is the authors’ opinion that an independent economic report should be commissioned to examine in detail the financial structure and future sustainability of the prevailing practice model. The issue of the gender pay gap highlighted in this study also requires significant attention.

## Conclusion

This study has highlighted several factors associated with the retention of veterinary employees, particularly in clinical practice, in Ireland. Veterinary employers should consider salary, working hours and the facilitation of a good work-life balance in order to successfully retain veterinary employees. The significant difference in salaries currently offered to male and female veterinary practitioners is an important finding of this study and warrants further investigation. Additionally, the finding that 33% of study respondents described themselves as likely to leave the veterinary profession in the next 5–10 years warrants further research to determine steps or measures that can be taken by the profession to tackle this issue and increase the long-term attractiveness of clinical veterinary practice in particular for registrants. There is an onus on leaders within the veterinary profession, and in relevant stakeholder groups within the wider public and agricultural sectors, to address these factors and look to safeguard the future sustainability of this essential and invaluable profession.

## Supplementary Information

**Additional file 1.**

**Additional file 2.**

**Additional file 3.**

**Additional file 4.**

## Data Availability

The datasets used and/or analysed during the current study are available from the corresponding author on reasonable request. Additional files are provided with the data in the manuscript.

## References

[1] Sheehan Quinn HLB. Veterinary Practice Survey Report 2021–2022. Pages 1-27. https://www.hlbsheehanquinn.ie/latest/veterinary-practice-survey-2021-22/. Accessed 21 Apr 2022.

[2] Veterinary Council of Ireland Annual Report 2021. Published March 2022. Pages 1-65. https://vci.ie/getmedia/5fbcd61e-938e-4976-87a9-58954cc156a3/VCI-Annual-Report-2021.pdf.

[3] Veterinary Ireland (2021). Classifieds. Vet Irel J.

[4] Veterinary Ireland (2016). Classifieds. Vet Irel J.

[5] Sheehan Quinn HLB. Veterinary Practice Survey Report 2019-2020. [internet]. Dublin 2020.

[6] Sheehan Quinn HLB. Veterinary Practice Survey Report 2020–2021 [internet]. Dublin; 2020. Available from: https://a.storyblok.com/f/70568/x/9f5ab2ef2c/veterinary-practice-survey-report-2020-2021-hlb-sheehan-quinn.pdf.

[7] Hagen JR, Weller R, Mair TS, Kinnison T (2020). Investigation of factors affecting recruitment and retention in the UK veterinary profession. Vet Rec.

[8] Adam K, Baillie S, Rushton J (2015). Retaining vets in farm animal practice: a cross-sectional study. Vet Rec.

[9] Vet Futures Project Board. Taking charge of our future. A vision for the veterinary profession for 2030 [Internet]. 2015. Available from: https://www.vetfutures.org.uk/resources/

[10] Navarre C, Daniels A, Johnston MO, Mathis C, Perrett T, Posey D, et al. Impact of Recruitment and Retention of Food Animal Veterinarians on the U.S. Food Supply. Counc Agric Sci Technol (CAST). 2020;(67):1-16. Available from: https://www.cast-science.org/wp-content/uploads/2020/03/CAST_IP67_Vet-Students.pdf.

[11] Achoui M, Mansour M (2007). Employee turnover and retention strategies: evidence from Saudi companies. Int Rev Business Res Papers.

[12] Iqbal A (2010). Employee turnover: causes, consequences and retention strategies in the Saudi organizations. Business Rev Cambridge.

[13] Mello JA (2011). Strategic human resource management.

[14] Federation of Veterinarians of Europe (FVE). Survey of the veterinary profession in Europe [internet]. Federation of Veterinarians in Europe. 2019. Available from: https://fve.org/cms/wp-content/uploads/FVE_Survey_2018_WEB.pdf

[15] Qualtrics software. Provo, UT: Copyright © 2019, Qualtrics; 2019.

[16] Veterinary Voices Ireland. Veterinary Voices Ireland [Internet]. Facebook.com. 2021 [cited 2021 Jul 31]. Available from: https://www.facebook.com/groups/vetvoicesireland/

[17] Progressive veterinary network. Progressive veterinary network (Facebook) [internet]. Facebook.com. 2021 [cited 2021 Jul 31]. Available from: https://www.facebook.com/ProgressiveVetNetwork/

[18] Progressive Veterinary Network (PVN). Progressive Veterinary Network [Internet]. https://pvn.ie/. 2021 [cited 2021 Jul 31]. Available from: https://pvn.ie/

[19] Muldoon, N. Registrar of the veterinary Council of Ireland, personal communications 2019 and 2022; www.vci.ie

[20] Krejcie RV, Morgan DW (1970). Determining sample size for research activities. Educ Psychol Meas.

[21] Microsoft Corporation. Microsoft Excel [Internet]. Microsoft Corporation; 2013. Available from: https://office.microsoft.com/excel

[22] R Core Team (2019). R: A language and environment for statistical computing [Internet].

[23] Wickham, H., François, R., Henry, L., & Müller K. Dplyr: A grammar of data manipulation [Internet]. 2020 [cited 2021 Jul 31]. Available from: https://cran.r-project.org/package=dplyr

[24] Offiah G, Murray F, Walsh C (2020). Doctor Retention in Ireland - Where Are the Failings That Prolong the Problem? Comment on “Doctor Retention: A Cross-sectional Study of How Ireland Has Been Losing the Battle”. Int J Heal Policy Manag.

[25] Veterinary Council of Ireland. Official Guidelines for New or Returning Graduates and their Employers [Internet]. Dublin; 2014 [cited 2021 Jul 31]. Available from: https://www.vci.ie/getmedia/741e4341-e48c-44d6-ac0c-216e5442c89a/Guidelines-New-Graduates-and-Returnees-11SEPT14.pdf?ext=.pdf

[26] Meehan MP, Bradley L (2007). Identifying and evaluating job stress within the Australian small animal veterinary profession. Aust Vet Pract.

[27] Kersebohm JC, Lorenz T, Becher A, Doherr MG (2017). Factors related to work and life satisfaction of veterinary practitioners in Germany. Vet Rec Open.

[28] Byrne JP, Conway E, McDermott AM, Matthews A, Prihodova L, Costello RW (2021). How the organisation of medical work shapes the everyday work experiences underpinning doctor migration trends: the case of Irish-trained emigrant doctors in Australia. Health Policy (New York).

[29] Haraldsson GD, Kellam J. Going Public: Iceland’s Journey To A Shorter Working Week [Internet] 2021. Available from: https://autonomy.work/wp-content/uploads/2021/06/ICELAND_4DW.pdf

[30] Villarroel A, McDonald SR, Walker WL, Kaiser L, Dewell RD, Dewell GA (2010). A survey of reasons why veterinarians leave rural veterinary practice in the United States. J Am Vet Med Assoc.

[31] Eurostat. Gender pay gap in unadjusted form [Internet]. Eurostat. 2021 [cited 2021 Jul 31]. Available from: https://ec.europa.eu/eurostat/databrowser/view/tesem180/default/table?lang=en

[32] Society of Practising Veterinary Surgeons. (SPVS) Salary survey [Internet]. 2020. Available from: https://spvs.org.uk/salary-survey-results/

[33] National Women’s Council of Ireland. Women and employment [internet]. National Women’s Council 2021 [cited 2021 Jul 31]. Available from: https://www.nwci.ie/discover/what_we_do/womens_economic_independence/women_and_employment

[34] The gender pay gap information act 2021 [internet]. Number 20 of 2021 Ireland; 2021. Available from: https://data.oireachtas.ie/ie/oireachtas/act/2021/20/eng/enacted/a2021.pdf.

[35] Yu ITS, Tse SLA (2012). Workshop 6 - sources of bias in cross-sectional studies; summary on sources of bias for different study designs. Hong Kong Med J.

[36] American Veterinary Medical Association (AVMA). Veterinary Salary Estimator for New Veterinarians [Internet]. 2019 [cited 2021 Jul 31]. Available from: https://myvetlife.avma.org/new-veterinarian/your-financial-health/veterinary-salary-estimator

[37] Organisation of working time act 1997. S.I. No. 20 / 1997 - Organisation of Working Time Act [Internet]. 20 of 1997 Ireland: Irish Statute Book; 1997 p. 1–12. Available from: http://www.irishstatutebook.ie/eli/1997/act/20/section/15/enacted/en/html#sec15

[38] Hämmig O (2018). Explaining burnout and the intention to leave the profession among health professionals - a cross-sectional study in a hospital setting in Switzerland. BMC Health Serv Res.

[39] Johnson JV, Lipscomb J (2006). Long working hours, occupational health and the changing nature of work organization. Am J Ind Med.

