# Supplementary material for: Factors affecting retention of veterinary practitioners in Ireland: a cross-sectional study with a focus on clinical practice
Source: Ir Vet J. 2022 Jun 7;75:13. doi: 10.1186/s13620-022-00222-9 (PMC9172024; doi:10.1186/s13620-022-00222-9)
Supplement: Supplementary file 1 — Additional file 1. [file 13620_2022_222_MOESM1_ESM.docx]

***Additional File 1: Questions included in the Survey***

| **Category** | **Variable Assessed** | **Question Asked** |
| --- | --- | --- |
| Demographics | Working in Republic of Ireland | Do you work as a veterinarian in the Republic of Ireland? (includes clinical and non-clinical roles which require a veterinary degree) |
|  | Registered With Veterinary Council of Ireland | Are you registered with the Veterinary Council of Ireland? |
|  | Gender | Are you male or female? |
|  | Age | Please enter your age |
|  | Location | Where are you currently working? |
|  | University Graduated | What university awarded your primary veterinary degree to you? - Selected Choice |
|  | Years Graduated | How many years ago were you awarded your veterinary degree? |
|  | Employment Status | Are you in : [Full time employment/Part time employment/Unemployed/Other] |
|  | Area Of Veterinary | In what area of the veterinary profession do you work in?  [Clinical practice/ Academia / Government veterinary service/ Research institution/Pharmaceutical industry / Other] |
|  | Employment Type | How would you describe your employment position?: [Associate Vet/Manager (Clinical director) / Practice Partner/Practice owner/Self employed/Full time locum/Unemployed] |
|  | Species Treating | You answered clinical practice to the last question. What species do you predominantly work with? [Mixed/Equine/Farm animal/Small animal/Other] |
| Remuneration | Salary | What is your current gross annual salary (Answer in Euro, number only e.g. 30,000)?  How satisfied or dissatisfied are you with your salary? |
| Working hours | Days per week worked | On average how many days a week do you work (including on call)? |
|  | Hours per week worked | On average how many hours do you work a week (including on-call)?  How satisfied or dissatisfied are you with your on call rota? |
|  | Out Of Hours (OOH) Rota | If you do on call, what is the rota: [Every night / 1 in 2 / 1 in 3 / 1 in 4 / 1 in 5 / 1 in 6 / Other / Do not do on-call]  Do you receive any of the following for on-call hours worked: [Over-time (On-call pay) / Time off in lieu / Both / Neither / Other]  How satisfied or dissatisfied are you with your hours of work? |
| Benefits, Terms & Conditions | Contract | Are you on a permanent or temporary contract?  Do you have a written contract? |
|  | Appraisals | Do you get regular staff appraisals? |
|  | Annual Leave | What is your paid annual leave entitlement? (Days)  Do you have to use your annual leave to attend CPD? |
|  | VCI Fees | Does your employer pay your VCI fees? |
|  | CPD Fees | Does your employer pay for your CPD?  You answered 'Yes' to the previous question. To follow on from this, what proportion of your CPD does your employer pay for? |
|  | Sick Leave | Are you entitled to sick leave? |
|  | Maternity/Paternity Leave | Are you entitled to maternity/paternity leave? |
|  | Benefits And Terms Satisfaction | How satisfied or dissatisfied are you with your benefits and terms and conditions (eg annual leave, expenses, CPD, etc)? |
| Career to date | No. of Jobs Worked | How many jobs have you had since qualifying? (Please give number of jobs) |
|  | Longest Duration Worked | What was the longest duration (in years) of employment in one job (excluding locums)? |
|  | Length of stay of last job | How long did you stay in your last job? |
|  | Career Prospects | What are your career aspirations? [Practice employee / Practice partner/ Practice owner/ Non-clinical role / Academia/ Research/Other] |
|  | Temporary Veterinary Inspector | Would you like to be able to join a meat factory panel as a Temporary Veterinary Inspector?  Do you think your income has suffered because you have not been able to supplement it with TVI meat factory shifts?  If the meat factory TVI panels open up again soon, would you rather be a contractor or an employee? |
|  | DAFM work | Would you consider applying for a job in the Department of Agriculture, Food and the Marine (DAFM)? |
|  | Brexit | Do you think Brexit will affect recruitment and/or retention of vets in Ireland? |
|  | Work Life Balance | How satisfied or dissatisfied are you with your current work life balance (i.e. that you have sufficient time for other pursuits such as family, exercise, hobbies, etc)? |
|  | Current Job | How satisfied or dissatisfied are you in your current job? |
| Future Career | Stay / Leave Ireland | How likely are you to stay in Ireland in the foreseeable future? |
|  | Stay / Leave Vet Profession | How likely are you to leave the veterinary profession in the next 5-10 years? |
|  | Stay / Leave Current Job | How likely is it that you will be looking for a new job in the next 2 years? |
|  | Reason For Leaving | If you are considering leaving your current position what is the main reason? Please pick the three most important factors. - Selected Choice |
|  | Stay / Leave Current Job | If you are planning on leaving your current position in the next 2 years what would you be looking to do? - Selected Choice |
|  | Become A Vet Again? | If you had your choice again, would you choose veterinary as a career? |
| Any Comments? | Text Analysis | This brings us to the end of the questions.  Thanks for your participation. If you would like to bring anything to our attention please feel free to put your comments here. |
