# Supplementary material for: Factors affecting retention of veterinary practitioners in Ireland: a cross-sectional study with a focus on clinical practice
Source: Ir Vet J. 2022 Jun 7;75:13. doi: 10.1186/s13620-022-00222-9 (PMC9172024; doi:10.1186/s13620-022-00222-9)
Supplement: Supplementary file 2 — Additional file 2. [file 13620_2022_222_MOESM2_ESM.docx]

***Additional File 2: Demographics of Study Population (n=370)***

| **Category (n = respondents answered)** | **Answer** | **%** | **n** |
| --- | --- | --- | --- |
| Consent Given*° | Yes  No  Answered  Missing data (not answered) | 100  0.0  -  - | 370  0  370  0 |
| Working in Ireland*° | Yes  No  Answered  Missing data | 100  0.0  -  - | 370  0  370  0 |
| Registered With VCI *° | Yes  No  Answered  Missing data | 100  0.0  -  - | 370  0  370  0 |
| Gender | Male  Female  Answered  Missing data | 32.1  67.4  100  - | 118  248  368  4 |
| Age | Mean (SD)  Answered  Missing data | 33.2 (7.9)  100  - | 364  6 |
| Location (Top 5) *¹ | Dublin  Cork  Galway  Tipperary  Meath  Answered  Missing data | 15.8  13.9  7.1  7.4  5.4  100  - | 58  51  26  27  20  367  3 |
| University Graduated (Top 5) | University College Dublin  Szent Istvan University, Budapest  University of Glasgow  University of Liverpool  University of Edinburgh  Rest of Europe  Answered  Missing data | 81.4  10.4  1.4  1.1  1.1  4.6  100  - | 298  38  5  4  4  17  366  4 |
| Years Graduated | Mean (SD)  Answered  Missing data | 8.7 (7.9)  100  - | 358  12 |
| Area Of Veterinary *² | Clinical practice  Government vet service  Academia  Pharmaceutical  Other  Answered  Missing data | 88.5  7.4  2.2  0.3  1.6  100  - | 324  27  8  1  6  366  4 |
| Employment position*³,ª | Associate Veterinarian/employee  Practice owner/partner/clinical director  Self employed/locum  Answered  Missing data | 78.3  14.6  7.1  100  - | 253  47  23  323  1 |
| Species Treating *³ | Mixed  Small animal  Farm animal  Equine  Answered  Missing data | 42.7  35.6  15.2  6.2  100  - | 138  115  49  20  322  1 |

**° If answered no, redirected to end of survey. *¹ If answered abroad, redirected to end of survey. *² Only seen if unemployed not selected in previous question. *³ If Clinical practice selected in ‘Area of Veterinary’ question. ª Multiple answer question*
