# Supplementary material for: Factors affecting retention of veterinary practitioners in Ireland: a cross-sectional study with a focus on clinical practice
Source: Ir Vet J. 2022 Jun 7;75:13. doi: 10.1186/s13620-022-00222-9 (PMC9172024; doi:10.1186/s13620-022-00222-9)
Supplement: Supplementary file 3 — Additional file 3. [file 13620_2022_222_MOESM3_ESM.docx]

***Additional File 3: Variables Significant Associated, on Univariate Analysis, with the Likelihood of Respondents Leaving their Current Job within 2 Years***

| ***Variables Negatively Associated with Likelihood of Leaving Current Job within 2 Years*** | ***Variable*** | ***P-value*** |
| --- | --- | --- |
|  | **Satisfaction with Current job** | **4.28E-13** |
|  | **Satisfaction with Hours Worked** | **4.67E-08** |
|  | **Satisfaction with Benefits** | **3.99E-09** |
|  | **Some CPD paid** | **0.000236** |
|  | **Rota 1 in 5** | **0.00707** |
|  | **Satisfaction with Out of Hours Work** | **0.005401** |
|  | **Satisfaction with Salary** | **0.00214** |
|  | **Maternity/Paternity Leave Provided** | **0.00324** |
|  | **Job allows Sick leave** | **0.0128** |
|  | **Position as Practice owner/partner/director** | **4.74E-07** |
|  | **Satisfied with Work-Life balance** | **3.90E-08** |
|  | **Unlikely to Leave Veterinary in 5-10 years** | **1.13E-09** |
|  | **Meat Factory TVI aspiration Yes** | **0.000651** |
|  | **Gender Male** | **0.000146** |
|  | **Area of Work: Government Veterinary Service** | **0.000371** |
|  | **Working in Counties Kildare & Meath** | **0.00237** |
|  | **Would not Consider joining DAFM** | **0.00345** |
|  | **Length last job 5 years or greater** | **0.000587** |
| **Variables Positively Associated with Likelihood of Leaving Current Job within 2 Years** | ***Variable*** | ***P-value*** |
|  | **Leaving to take a break for family or travel** | **0.0168** |
|  | **Not given annual leave or CPD allowance** | **0.0206** |
|  | **Leaving reason family and work-life balance** | **0.0416** |
|  | **No strong feelings about remaining in Ireland** | **0.0427** |
| **Quantitative Variables with Varying Effects on Likelihood of Leaving Current Job within 2 Years** | ***Variable*** | ***P-value*** |
|  | **Years Qualified** | **8.92E-08** |
|  | **Age** | **3.27E-07** |
|  | **Log_10_salary** | **1.93E-06** |
|  | **No. jobs since qualified** | **0.000134** |
|  | **Longest job stay** | **0.0499** |
