# Supplementary material for: Factors affecting retention of veterinary practitioners in Ireland: a cross-sectional study with a focus on clinical practice
Source: Ir Vet J. 2022 Jun 7;75:13. doi: 10.1186/s13620-022-00222-9 (PMC9172024; doi:10.1186/s13620-022-00222-9)
Supplement: Supplementary file 4 — Additional file 4. [file 13620_2022_222_MOESM4_ESM.docx]

***Additional File 4: Variables Significantly associated with Log_10_salary on Univariate Analysis***

| **Variables Associated with Higher Log_10_salary** | **Variable** | **P-value** |
| --- | --- | --- |
|  | Years Qualified | 2.00E-16 |
|  | Age | 7.85E-15 |
|  | Longest job stay | 3.47E-14 |
|  | Length last job 5 or greater | 9.03E-05 |
|  | New job in 2 years Unlikely | 1.70E-07 |
|  | No. jobs since qualified | 0.00756 |
|  | Gender | 7.36E-11 |
|  | Area Government Veterinary Service | 7.08E-07 |
|  | Satisfaction with Salary | 1.59E-06 |
|  | Satisfaction with Job Benefits | 0.000127 |
|  | Hours per week | 0.000411 |
|  | Days per week | 0.000861 |
|  | Maternity/Paternity Leave available in Job | 0.00204 |
|  | Sick leave available in Job | 0.00684 |
|  | Paid leave days | 0.0254 |
|  | Satisfied with Current Job | 0.0318 |
|  | Leaving the profession reason Retirement | 0.000186 |
|  | Position Practice owner/partner/director | 0.00165 |
|  | Working in Counties Kildare&Meath | 0.00294 |
|  | Regular Staff Appraisal Given | 0.00885 |
|  | Opinion that Brexit is not likely to affect recruitment and retention | 0.009 |
|  | Rota 1 in 3 | 0.0257 |
| **Variables Associated with Lower Log_10_salary** | **Variable** | **P-value** |
|  | Employment Part-time | 3.03E-08 |
|  | Unlikely to stay in Ireland | 0.00252 |
|  | Opinion that Income suffered by not having a TVI Shift | 0.0176 |
|  | Aspiration for Meat Factory TVI Work | 0.0228 |
